# Dynamic balance control in healthy young women during stair descent: a plantar pressure-based study

**DOI:** 10.3389/fbioe.2025.1517471

**Published:** 2025-01-29

**Authors:** Ruiqin Wang, Jinfeng Cao, Haoran Xu, Panjing Guo, Yumin Li, Yuyi Fan, Yunfei Gui, Leqi Li, Roger Adams, Jia Han, Jie Lyu

**Affiliations:** ^1^ Department of Orthopedics, Jinshan District Central Hospital Affiliated to Shanghai University of Medicine and Health Sciences, Shanghai, China; ^2^ College of Rehabilitation Sciences, Shanghai University of Medicine and Health Sciences, Shanghai, China; ^3^ Periodicals Agency, Shanghai University, Shanghai, China; ^4^ College of Medical Instruments, Shanghai University of Medicine and Health Sciences, Shanghai, China; ^5^ Research Institute for Sport and Exercise, University of Canberra, Canberra, ACT, Australia

**Keywords:** stair descent, females, landing strategy, COP, kinematic parameters, landing strategy

## Abstract

**Background:**

Women are more likely to fall or even die when the ladder falls, which seriously affects the quality of daily life. It is necessary to better understand the plantar mechanism of the ladder falls and put forward reasonable suggestions.

**Method:**

Twenty healthy young women volunteered to participate in the experiment. The study used the F-scan plantar pressure to explore the difference in the plantar pressure in the dominance of the leading foot across four step descent height conditions. The landing strategy employed was recorded during the experiment. The Center of Pressure (COP), along with its medial-lateral (ML) and anterior-posterior (AP) adjustment velocities, and the *V*_COP_, *R*_COP-ML_, and *R*_COP-AP_ were analyzed.

**Result:**

With an increase in the step height, significant enhancements were observed in the *V*_COP-ML_ (p < 0.001), *V*_COP-AP_ (p < 0.001), *R*_COP-ML_ (p < 0.001), and *R*_COP-AP_ (p < 0.001) during landing. There was no significant difference in the kinematic parameters of plantar pressure during stair descent, regardless of whether the dominant foot or non-dominant foot was the leading foot.

**Conclusion:**

This study found that among young women, an increase in step height during descent significantly affected the plantar pressure and led to greater COP adjustment in the directions of ML and AP, increasing the risk of injury. At a step height of 5 cm, the first choice of the landing strategy for female subjects began to change from the hindfoot to the forefoot. Although there were no significant differences in plantar pressure data and landing strategies between subjects using the dominant side and nondominant side as the forefoot, the dominant side forefoot exhibited better postural balance control than the nondominant side forefoot.

## 1 Introduction

The descent of stairs is one of the most common activities in workplaces, homes, and communities. Falls incurred during stair descent have been reported as the third leading cause of injuries, accompanied by a higher mortality rate compared to other fall types (Ragg et al., 2000; Cai et al., 2023). These falls, in addition to their frequency, often result in ankle sprains, with previous studies indicating that approximately one-quarter of all hospital-treated ankle sprains stem from stair descent (Waterman et al., 2010). Postural balance control, defined as the capacity to swiftly adjust one’s center of gravity to maintain posture stability amidst static, dynamic, or sudden external forces, is paramount. Consequently, the purpose of this paper is to explore the underlying mechanisms of postural balance control of human body during stair descent across different environments and to put forward practical recommendations to reduce injuries during stair descent, thereby enhancing both individuals’ life quality and community safety.

Stair descent-related falls pose a significant threat to community safety, transcending age boundaries and affecting both the elderly and young alike. Notably, among adults aged 18–24 in the United States, the cumulative cost of fall-related injuries exceeded $7 billion (Verma et al., 2016). Given their heightened activity levels, young people experience a greater frequency of falls. Furthermore, adult women consistently exhibit a higher propensity for falls than adult men. Research (Talbot et al., 2005) has shown that, among young adults aged 20–45, women experience falls more frequently than men (20% vs. 17%) and suffer injuries more often (81% vs 61%). Specifically, 48% of falls among young adults are closely related to stair descent (Cho et al., 2021). Compared to men, women were more likely to fall on stairs (15% vs. 10%) (Cho et al., 2021). Additionally, gender disparities are evident in lower limb functional measures, with men typically demonstrating superior muscle strength (Barber-Westin et al., 2006; Danneskiold-Samsøe et al., 2009), muscle activation patterns (Flaxman et al., 2014; Bencke and Zebis, 2011), and joint laxity (Davey et al., 2019). Therefore, exploring gender-specific injury mechanisms during stair descent is imperative.

During stair descent, accidents frequently occur during the transition from stairs to level ground. Research (Francksen et al., 2022; Duckham et al., 2013) has indicated that over 30% of stair-related accidents occur precisely during this transition phase, where even a single step or curb could lead to serious falls. Previous studies (Cluff and Robertson, 2011; Sie et al., 2022) have predominantly focused on lower limb mechanics during continuous stair descent. In light of research (Andriacchi et al., 1980) examining the differences in lower limb mechanics between continuous descent and the transition to level walking, it is evident that the mechanics of continuous stair descent cannot be universally applied to the step-to-level transition. Few studies have explored the biomechanics of the lower extremity in the transition between a single step and a flat surface, but the hazards of falls and injuries occurring during this transition should not be underestimated. Consequently, the step-to-level transition emerges as a pivotal aspect requiring focused attention to understand the mechanisms underlying falls and ankle injuries.

The foot supports the majority of the body weight during human activities and serves as a vital organ of the body. The plantar pressure distribution varies under different conditions (Razak et al., 2012), providing feedback on the structural composition of the human body and the control of postural balance (Li et al., 2023). Furthermore, analyzing plantar pressure parameters holds significant potential for the development of wearable footwear devices in the future. Plantar pressure measurement is a technique used to capture the distribution of plantar pressure and the center of pressure (COP) data during static and dynamic states. This technique enables the acquisition of key biomechanical parameters related to human kinematics and dynamics, playing a crucial role in gait analysis, clinical diagnosis, and functional assessment of lower limb musculoskeletal disorders.

Two pivotal factors that may influence postural balance control during stair descent are the step height and the dominance of the leading foot (i.e., dominant vs non-dominant). Postural balance control is facilitated by a cohesive feedback loop involving the interplay of vision, proprioception, and vestibular sense, all contributing to the maintenance of body balance. Impairments in any of the above three parts can affect the balance regulation capability of the human body (Peterka, 2002). As the step height increases, significant changes in lower limb proprioception are observed (Gerstle et al., 2017), potentially compromising posture control during stair descent. Additionally, studies (Gerstle et al., 2017; Gerstle et al., 2018; van Dieën and Pijnappels, 2009) have shown that an increased step height may lead to more complex lower limb muscle activity during stair descent and significantly alter landing strategies and ankle joint kinematics. In addition, use of the dominant or non-dominant side of the lower limbs can have a significant impact on postural balance control. Studies on Body Tracking Tests (BTT) reveal that ankle strategies and balance control in the AP direction were significantly influenced by the choice of leading foot (Yoshida et al., 2013). The dominant lower limb was more crucial for weight support and functional activities, possibly affecting balance (Hoffman et al., 1998; Alonso et al., 2011). Therefore, studying the effects of step height and the dominance of the leading foot during stair descent is valuable for understanding balance control mechanisms.

Accordingly, this study focused on young women to investigate the impact of two factors, i.e., the step height and the dominance of the leading foot (dominant vs non-dominant), on balance control and landing strategies employed during stair descent. Plantar pressure measurement equipment was utilized to assess biomechanical characteristics and landing strategies across four different heights. Our hypotheses stipulate: 1) An increase in the step height would diminish balance control and affect landing strategies during stair descent; 2) The dominance of the leading foot (dominant or non-dominant) modulates balance and landing strategies during stair descent.

## 2 Materials and methods

### 2.1 Participants

The recruitment period of this experiment was from March 15, 2023 to June 15, 2023. Subjects who met the inclusion and exclusion criteria were selected and signed written informed consent. This study, approved by the Ethics Committee of Shanghai Sport University (No.: 102772021RT073), involved 20 young women with a mean age of 21.30 ± 0.98 years, a height of 164.30 ± 4.64 cm, a weight of 57.18 ± 11.82 kg, and a BMI of 21.17 ± 4.34. Prior to conducting the experiment, the minimum number of subjects required was calculated based on the G-power. The statistical method was a repeated measures ANOVA with a higher effect size set at 0.4 and an efficacy value of 0.8, resulting in a minimum sample size of 13 subjects. All participants possessed shoe sizes ranging from 36 to 39 and were right-footed, as determined by the Chinese version of the Waterloo Footedness Questionnaire (Yang et al., 2018). Informed consent was obtained from all participants after a thorough explanation of the experimental requirements. The research was conducted in accordance with the guidelines outlined in the Declaration of Helsinki. However, a total of 22 female data were taken in our study, with data of 20 subjects ultimately included in the analysis.

Inclusion Criteria: a) women aged between 18 and 25 years, b) no history of musculoskeletal or neurological disorders that may affect postural balance control, and c) ability to walk and descend stairs independently without the aid of assistive devices. Exclusion Criteria: a) persistent pain or a history of surgery in the hip, knee, ankle, or foot within the past 6 months, b) poor compliance, preventing completion of the experiment, and c) allergies to materials involved in the experiment.

### 2.2 Procedure

#### 2.2.1 Pre-test preparation

**FIGURE 1:**
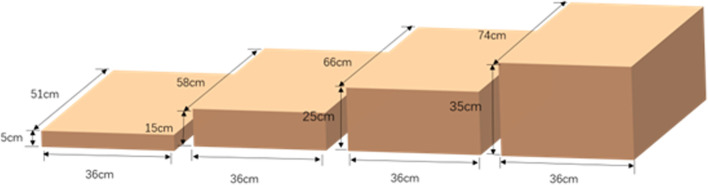
Illustration of step heights of 5 cm, 15 cm, 25 cm, and 35 cm.

The experiments were conducted in a noise-controlled environment to mitigate auditory disturbances. The primary equipment comprised four steps of varying heights: 5 cm, 15 cm, 25 cm, and 35 cm (dimensions in cm: 51×36×5 , 58×36×15 , 66×36×25 , and 74×36×35 , respectively). The four types of step heights include the doorstep height of 5 cm, the common residential staircase height of 15 cm, the step height of 25 cm recommended by the U.S. Federal Highway Administration guidelines (Gerstle et al., 2017), and the typical bus pedal height of 35 cm. The height of the four steps is shown in Figure 1. All subjects followed the same step height sequence from low to high. The four step heights are designed to encompass common roadside (5 cm) and building stairs (15 cm) in daily life, as well as the 25 cm step height issued by the US Federal Highway Administration, and the 35 cm step height to simulate challenging steps encountered by healthy adults

Prior to the testing day, participants were instructed to avoid high-intensity activities and to walk freely with the instrumented insoles inserted for 5 min to ensure a natural gait. For each participant, the F-Scan system was calibrated twice as per the manual to ensure the equipment work properly. Essential details such as name, height, weight, and date were recorded before beginning the experimental briefing and guidance on the tasks.

#### 2.2.2 Equipment

**FIGURE 2:**
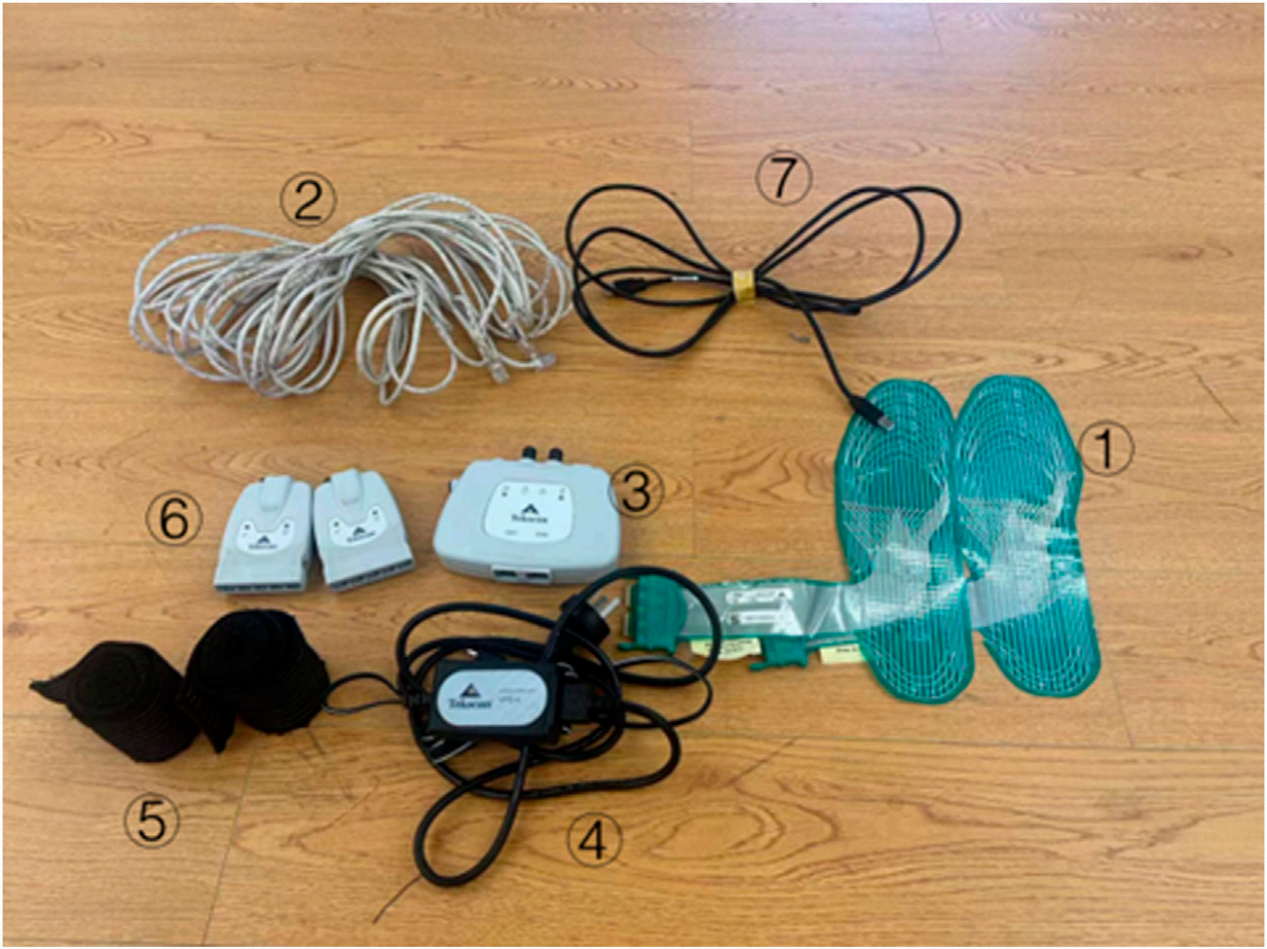
F-Scan plantar pressure system hardware schematic.

The two most commonly used plantar pressure insole systems in the market are the F-Scan system of TEKSCAN (USA) (Chen and Yu, 2005) and the Pedar-X system of Novel (Germany) (Ma, 2002). In this experiment, the F-Scan plantar pressure system of TEKSCAN (United States) was employed. The F-Scan plantar pressure system (Tekscan, Boston, MA, United States) was used to collect data. This system, featuring a customizable insole with four sensors per square centimeter and a sampling rate of 50 Hz, records real-time plantar pressure distribution. In order to minimize the displacement between the shoe and the insole, participants wore standardized cotton socks on bare feet and fixed the insole and cotton socks with double-sided tape (Guo et al., 2023). Figure 2 shows the F-Scan device. This figure depicts the following: ① the plantar pressure insole, ② the CAT5E cable connector, ③ the VersaTek two-port hub, ④ the power cord, ⑤ the fixed ankle bandage, ⑥ the VC-1 VersaTek converter, and ⑦ the USB data connection cable.

#### 2.2.3 Testing procedure

Upon completion of the preparatory steps, all participants were instructed to stand in a consistent position at the top of the step, with their feet relaxed and their eyes directed forward for a natural descent. Upon landing with the first foot, the non-first foot followed, and participants remained in a standing position for 5 s to enable the recording of the sole pressure. The experiment encompassed 8 combinations, determined by the step height (5 cm, 15 cm, 25 cm, and 35 cm) and the dominance of the leading foot (dominant or non-dominant) (Guo et al., 2024). This procedure was repeated three times for each condition, with the F-Scan system capturing the data. Upon completion of the experiment, the data were exported into CSV files using the F-Scan plantar pressure system for kinematic data. After each participant completes the F-Scan plantar calibration, the calibrated parameters of the left and right feet are compared. If the discrepancy is too large, recalibration is performed. If the discrepancy remains significant after the second calibration, the participant’s experiment is discontinued. Upon completion of each experiment, the researchers verify the completeness of the plantar pressure data and ensure that the foot does not extend beyond the insole’s boundaries. If such issues are detected, the experiment for that participant is repeated.

#### 2.2.4 Data processing

Plantar pressure data, including the center and distribution, were analyzed to assess postural stability. Python (Pycharm Community Edition 2022.2, JetBrains s.r.o., Prague, Czech Republic) algorithms were utilized to process the data and extract kinematic parameters. These parameters include the landing strategies across different step heights and a series of parameters related to the center of pressure such as COP-ML adjustment velocity (mm/s), COP-AP adjustment velocity (mm/s), COP adjustment velocity (mm/s), 95% confidence circle area ( ${\text{mm}}^{2}$ ), ML range (mm), and AP range (mm). The 95% confidence circle area represents the area where the sole of the foot oscillates during stair descent. A larger value of this parameter indicates greater variation in the participant’s plantar COP, suggesting a higher challenge to postural balance control and an increased risk of falling.

The above measures were obtained through a customized Python program incorporating relevant formulas (Guo et al., 2023), as detailed in Table 1.

**TABLE 1:** Formulas related to kinematic parameters.

Kinematic parameters	Abbreviation	Formula
COP-ML adjustment velocity	*V*_COP-ML_	$COP-ML\;adjustment\;velocity\left(m/s\right)=1/T\sum_{n-1}^{N-1}\left|\left[ML\left[n+1\right]-ML\left[n\right]\right]\right|$
COP-AP adjustment velocity	*V*_COP-AP_	$COP-AP\;adjustment\;velocity\left(m/s\right)=1/T\sum_{n-1}^{N-1}\left|\left[AP\left[n+1\right]-AP\left[n\right]\right]\right|$
COP adjustment velocity	*V*_COP_	$COP\;adjustment\;velocity\left(m/s\right)=1/T\sum_{n-1}^{N-1}{\left[{\left(\left[AP\left[n+1\right]\right]-AP\left[n\right]\right)}^{2}+{\left(ML\left[n+1\right]-ML\left[n\right]\right)}^{2}\right]}^{1/2}$
95% confidence circle area		$Mean\;Distance=1/N\sum_{n=1}^{N}{\left[AP{\left[n\right]}^{2}+ML{\left[n\right]}^{2}\right]}^{1/2}$
		$RMS\;Distance={\left[1/N\sum_{n=1}^{N}\left[AP{\left[N\right]}^{2}+ML{\left[N\right]}^{2}\right]\right]}^{1/2}$
		$95\%\;confidence\;circle\;area=\pi {\left(MDIST+1.645{\left[{RDIST}^{2}-{MDIST}^{2}\right]}^{1/2}\right)}^{2}$
ML range	*R*_COP-ML_	$ML\;range=\max_{1\leq n\leq m\leq N}\left|ML\left[n\right]-ML\left[m\right]\right|$
AP range	*R*_COP-AP_	$AP\;range=\max_{1\leq n\leq m\leq N}\left|AP\left[n\right]-AP\left[m\right]\right|$
Maximum swing	*S*_max_	$Maximum\;swing=\max_{1\leq n\leq N-1}{\left[{\left(AP\left[n+1\right]-AP\left[n\right]\right)}^{2}+{\left(\left[ML\left[n+1\right]\right]-ML\left[n\right]\right)}^{2}\right]}^{1/2}$
Minimum swing	*S*_min_	$Minimum\;swing=\min_{1\leq n\leq N-1}{\left[{\left(AP\left[n+1\right]-AP\left[n\right]\right)}^{2}+{\left(ML\left[n+1\right]-ML\left[n\right]\right)}^{2}\right]}^{1/2}$

Where, it is assumed that the recorded COP trace contains N data points, sampled at a constant frequency F_S. T represents the total duration of the signal in seconds, i.e.: $T=\frac{N}{F_{S}}$ .The n and m ( 1≪n≪N,1≪m≪N ) in the formula are the coordinates from back to forward on the AP axis and from left to right on the ML axis.

### 2.3 Statistical analysis

Data analysis was performed using SPSS (version 27.0; IBM) and Excel 2016 (Microsoft, Redmond, Washington, United States), with graphics generated by Origin2018 software (Origin Lab Corporation, Northampton, MA, United States). Data that followed a normal distribution were presented as mean ± standard deviation (SD). For data confirmed to follow a normal distribution after using the Kolmogorov-Smirnov test, a 2 × 4 repeated measures analysis of variance (ANOVA) was employed. For data deviating from a normal distribution, a generalized linear model was used with Bonferroni corrections for multiple comparisons. The experimental data of the plantar pressure center in this paper shows approximate normality, allowing for the application of the Grubbs criterion to detect outliers in the data. Subsequently, any outliers identified through this method can be removed. The significance level (α) was set at 0.05. A P-value of less than 0.05 indicates that the difference is statistically significant, while a P-value of less than 0.001 indicates an extremely significant statistical difference.

## 3 Results

### 3.1 Foot landing strategy

During the experiments, participants were instructed to land on steps of varying heights using either their dominant or non-dominant foot as the leading foot. When the step height was 5 cm, 50% (10 out of 20) of the participants adopted a forefoot landing strategy. As the step height increased to 15 cm, 90% (18 out of 20) of the participants chose to land on the forefoot first. For step heights of 25 cm and 35 cm, all participants (20 out of 20) employed the forefoot landing strategy consistently. Regardless of the height of the step, the probability of selecting the forefoot landing strategy with the dominant foot or non-dominant foot as the leading foot remains the same.

### 3.2 Plantar pressure center during stair descent

This study examined the effects of varying step heights and the dominance of the leading foot on the COP parameters at the plantar pressure center using analysis of variance. Additionally, the interaction between these two factors was explored. The findings revealed no statistically significant interaction between the step height and the dominance of the leading foot. Table 2 presents the results and significance level of all relevant parameters of COP. Only the area of the 95% confidence circle of the dominant foot compared with the non-dominant foot was statistically significant (p = 0.013), as the mean value for the non-dominant foot (6,249.01 mm^2^) was greater than that of the dominant foot (5,223.35 mm^2^). No other notable differences emerged due to the leading foot factor. Although the values for the non-dominant foot were greater than those for the dominant foot in the COP adjustment velocity and AP range indicators at 5 cm, 15 cm, and 25 cm step heights, the leading foot factor was not associated with significant differences. Similarly, for the AP adjustment velocity indicator, the non-dominant foot showed marginally higher values across all heights, yet these were not statistically significant.

**TABLE 2:** Comparison of COP parameters of plantar pressure during stair descent across different step heights and the dominance factor of the leading foot.

	5 cm step		15 cm step		25 cm step		35 cm step		*P* (height)	*P* (leading foot)
	N	D	N	D	N	D	N	D		
*V*_COP-ML_ (mm/s)	9.77 ± 1.89	10.90 ± 2.14	12.03 ± 1.84	13.51 ± 3.40	14.85 ± 2.82	14.13 ± 3.73	16.70 ± 2.73	17.32 ± 5.13	<0.001	0.206
*V*_COP-AP_ (mm/s)	55.12 ± 11.64	50.09 ± 8.93	60.23 ± 13.03	55.15 ± 12.04	66.04 ± 12.30	63.22 ± 10.54	77.63 ± 11.98	75.99 ± 14.6	<0.001	0.057
*V*_COP_ (mm/s)	57.55 ± 11.42	53.29 ± 8.86	63.79 ± 12.95	59.58 ± 12.54	70.74 ± 12.43	67.39 ± 10.36	82.23 ± 11.7	81.07 ± 15.14	<0.001	0.091
95% confidence circle area (mm^2^)	4,546.70 ± 2,531.80	3,673.98 ± 1895.43	5,366.30 ± 1794.34	4,451.46 ± 2,165.48	7,043.09 ± 3,420.52	5,619.20 ± 1930.27	8,039.93 ± 3,737.14	7,148.78 ± 2,194.9	<0.001	0.013
*R*_COP-ML_ (mm)	18.11 ± 6.60	18.85 ± 4.90	21.64 ± 6.52	23.80 ± 6.11	27.05 ± 9.22	23.40 ± 5.78	28.53 ± 5.96	28.21 ± 7.56	<0.001	0.802
*R*_COP-AP_ (mm)	116.06 ± 18.30	109.13 ± 18.38	118.33 ± 21.93	110.84 ± 21.52	123.27 ± 17.46	121.86 ± 19.84	132.52 ± 19.64	137.13 ± 17.8	<0.001	0.362
*S*_max_ (mm)	43.50 ± 16.02	47.43 ± 20.36	41.35 ± 18.93	36.96 ± 10.33	43.01 ± 10.396	43.26 ± 14.7	47.93 ± 15.61	46.68 ± 14.95	0.682	0.890
*S*_min_ (mm)	9.90E-03 ± 4.74E-03	1.09E-02 ± 5.27E-03	1.12E-02 ± 6.08E-03	1.17E-02 ± 3.92E-03	1.02E-02 ± 7.74E-03	1.29E-02 ± 4.35E-03	1.19E-02 ± 5.45E-03	1.36E-02 ± 8.23E-03	0.010	0.116

N: Non-Dominant, D: dominant.

Analysis of the plantar pressure data across the four different step heights as height increased revealed significant differences (p < 0.001) in several parameters: *V*_COP-ML_ (mm/s), overall *V*_COP_ (mm/s), 95% confidence circle area (mm^2^), *R*_COP-ML_ (mm), and *R*_COP-AP_ (mm). In contrast, the other two indices, *S*_max_ (mm) and *S*_min_ (mm) were not significantly affected by the changes in step height.

## 4 Discussion

This study investigated changes in the biomechanical attributes of the plantar surface of the dominant and non-dominant feet in young women during stair descent across varying step heights and under different leading foot conditions. The kinematic data revealed that both the landing strategy and the plantar pressure center during stair descent were closely associated with the step height. However, no significant effects on the landing strategy were discernible when comparing the use of dominant versus non-dominant foot as the landing foot.

### 4.1 Landing strategy

In normal flat gait, where the step height difference is 0, the contact between the sole and the ground during each step typically involves the back-foot touch strategy (Freedman and Kent, 1987). When the height difference increased from 0 to 5 cm, the subjects’ bodies faced few challenges of potential energy and balance control due to the minor drop. However, the results of this paper showed that 50% of female subjects still opt for the forefoot touch strategy to manage body potential energy and maintain body balance. Studies have shown (van Dieën et al., 2008; Van Der Linden et al., 2007) that even with a 5 cm height difference, subjects tend to choose the toe touch strategy when they cannot observe the height difference during step descent. During the experiment, the subjects could not see the height difference when looking ahead, which was consistent with the results of this study. Compared with the flat-bottom gait strategy, when the step descent height was 5 cm, the female subjects began to change from the back-foot approach to the front-foot approach.

At a step height of 15 cm, 90% of the participants opted for a forefoot landing strategy (Freedman and Kent, 1987). When the step height was increased to 25 cm and 35 cm, all participants switched their landing strategy from hindfoot to forefoot contacting the ground. When descending high steps, a forefoot landing augments the load on the ankle joint, potentially surpassing safe thresholds and predisposing individuals to ankle sprains or falls. Descending a step increases the potential energy due to the COM, and significantly raises the torque on the knees and ankles (Silverman et al., 2014). It has been noted that (Jeon et al., 2021) regardless of the landing strategy, joints, ligaments, and muscles have to absorb the potential energy caused by the COM changes. The process of descending a step can be divided into three phases: weight acceptance, forward continuation, and controlled descent (Zachazewski et al., 1993). During both weight acceptance and forward progression, ankle torque is higher with the forefoot strategy compared to the hindfoot strategy (Demers et al., 2021). Research has indicated that during the transition from a step to a flat surface, the potential energy absorbed by the ankle joint (van Dieën et al., 2008) is three times greater with the forefoot landing than with the heel landing. Therefore, choosing a forefoot landing strategy for higher step descents would allow subjects to better manage the balance challenges posed by height descents but would also increase ankle loading, with the risk of ankle sprains or falls when the tolerance threshold is exceeded. Importantly, all participants in this study were healthy individuals without any pre-existing lower limb injuries. Although the ankle joints of healthy individuals may be able to withstand the load brought by the forefoot touching the ground during descent from high steps, preventing unstable posture control or falls, the potential for accidents remains unmitigated.

As the height escalates to 20 cm, the adoption of the forefoot strategy during stair descent becomes increasingly prevalent (Freedman and Kent, 1987; van Dieën et al., 2008). In this paper, the proportion of participants using the forefoot strategy increased from 50% at 5 cm to 90% at 15 cm. Initially, we speculated that the reason for choosing the forefoot strategy at a lower height might be related to the height and leg length of the participants, and those with shorter height and leg length might change from the hindfoot strategy to the forefoot strategy at a lower step, so as to better control their postural balance and absorb the kinetic energy brought by the descent. However, it was found that there was no significant relationship between landing mode and anthropometric factors. This observation could be attributed to the experiment setup, where the participants gazed ahead, deprived of the ability to visually assess the step heights, potentially eliciting a degree of anxiety. Consistent with our findings, researchers in a previous study (Van Der Linden et al., 2007) hypothesized that the participants who could not see the difference in height would use a forefoot strategy when descending the steps, even for steps as low as 5 cm.

### 4.2 Plantar pressure parameter

The results presented herein indicated that COP-related parameters such as *V*_COP-ML_, *V*_COP-AP_, overall *V*_COP_, *R*_COP-ML_, and *R*_COP-AP_ all increased with the step height during stair descent. Compared to walking on flat ground, actions such as descending stairs and stepping off curbs involve a vertical descent of the center of mass (COM) because the body’s center of gravity shifts downward relative to the support surface, facilitating the smooth transfer of the COM. During stair descent, the COM is adjusted horizontally by leaning the body forward or backward to maintain balance, particularly at higher steps where the body tends to lean forward more to mitigate the impact of descent (Gosine et al., 2021). The COP, being the projection of the COM onto the support surface (Kilby et al., 2016), exhibits analogous patterns of variation. Consequently, as step height increased, participants adjusted the COM forward or backward tilt, as reflected in the COP, leading to increased COP adjustment velocities and ranges in the AP and ML directions. This aligns with our findings and previous studies indicating that the range of foot posture swings in the ML direction was associated with an increased risk of falling (Eckardt and Rosenblatt, 2018; Krishnan et al., 2013). During challenging daily activities, foot control was more prone to sway in the ML direction, affecting postural balance control. High *V*_COP_ and the swing range of COP are regarded as important indicators of balance instability and risk when falling, and the greater the COP value, the greater the postural swing, and thus the lower postural stability (Maki et al., 1994; Fernie et al., 1982). This study found that the increase in step height made the postural control of steps more challenging, augmenting the range of postural swing in the ML direction and increasing the risk of falling due to postural instability. These findings support hypothesis 1 of this paper, which states that heightened step heights diminish lower limb stability and balance when touching the ground. Walking down steps or stairs, as a common dynamic activity in daily life, may offer a more insightful glimpse into balance mechanisms than conventional balance tests such as bipedal standing, unipedal standing, or horizontal ground walking. From a kinematic perspective, as step height rises, the forward distance of the COM on the stairs becomes longer, accelerating the COP in the ML and AP directions, which reflects the instability of the step descent and increases the risk of injury and falling.

Comparing the plantar pressure parameters with the dominant and the non-dominant sides as the leading foot, the results of this study underscore a significant difference exclusively in the 95% confidence circle area parameters. Specifically, the 95% confidence circle area value of the dominant side as the leading foot was significantly lower than that of the non-dominant side as the leading foot. According to the index formula used in this paper, the 95% confidence circle area represents the plantar swaying area when walking down the steps. The smaller the value, the more stable the posture (Maki et al., 1994). The results also showed that the *V*_COP_, *R*_COP-ML_, and *V*_COP-AP_ of the dominant foot were lower than those of the non-dominant foot at 5 cm, 15 cm, and 25 cm step heights, although there was no significant difference in other COP-related indexes. According to the previous description, the higher the COP parameter value, the worse the postural stability. Comparing the dominant lower limb side with the non-dominant side as the leading foot during stair descent is important for understanding postural balance control. The results here support hypothesis 2: “The dominance of the leading foot (dominant or non-dominant) modulates balance and landing strategies during stair descent.” which is consistent with previous research (Hoffman et al., 1998; Alonso et al., 2011).

### 4.3 Limitations and future directions

This study focused exclusively on healthy young women, thus necessitating caution in generalizing the results to broader populations. Our research team plans to explore the stair descending characteristics of women across the entire age spectrum in future work, with the aim of further determining whether the findings of this study are applicable to other age groups or populations with balance disorders. Additionally, this experiment primarily investigated the influence of step height and foot dominance on biomechanics during stair descent. Other factors that may affect the descent strategy, such as participant height, lower limb strength, and environmental conditions, were not considered in this study. We intend to incorporate them into our subsequent research.

## 5 Conclusion

The results of this study indicate that among young female participants, the height factor during stair descent significantly influences the plantar pressure data of the subjects, with the increase in step height leading to notably larger adjustment speed and swing amplitude of the subjects’ COP in the ML and AP directions, thereby elevating the risk of injury and falls. At a step height of 5 cm, the first choice of the landing strategy for female subjects changed from the hindfoot to the forefoot. Although there was no significant difference in the plane pressure data of the two groups of subjects during stair descent, the postural balance control of the leading foot on the dominant side was stronger than the leading foot on the non-dominant side, and this factor had no influence on the choice of landing strategy.

It is suggested that young women choose the dominant side as the leading foot during stair descent to have a better control of postural balance control. As the step height increases, the risk of falling also rises. Therefore, it is recommended that the design height of daily stairs be kept within a reasonable range and minimized as much as possible. It is also recommended that the female population optimize ankle flexibility to reduce the risk of ankle sprains and falls associated with choosing a forefoot touchdown strategy.

## Data Availability

The raw data supporting the conclusions of this article will be made available by the authors, without undue reservation.
